# Prognostic implication and immunotherapy response prediction of a ubiquitination-related gene signature in breast cancer

**DOI:** 10.3389/fgene.2022.1038207

**Published:** 2023-01-04

**Authors:** Yangyang Guo, Qiaoqiao Chen, Yingjue Zhang, Xu Cheng, Kenan Cen, Ying Dai, Yifeng Mai, Kai Hong

**Affiliations:** ^1^ Department of Thyroid and Breast Surgery, Ningbo First Hospital, Ningbo, China; ^2^ Department of Thyroid and Breast Surgery, Ningbo Hospital of Zhejiang University, Ningbo, China; ^3^ Reproductive Medicine Center, The Affiliated Drum Tower Hospital of Nanjing University Medical School, Nanjing, China; ^4^ Key Laboratory of Reproductive Dysfunction Management of Zhejiang Province Assisted Reproduction Unit, Department of Obstetrics and Gynecology, Sir Run Run Shaw Hospital, Zhejiang University School of Medicine, Hangzhou, China; ^5^ Department of Molecular Pathology, Division of Health Sciences, Graduate School of Medicine, Osaka University, Suita, Japan; ^6^ Taizhou Hospital of Zhejiang Province Affiliated to Wenzhou Medical University, Taizhou, China; ^7^ The Affiliated Hospital of Medical School of Ningbo University, Ningbo, China

**Keywords:** breast cancer, ubiquitination, immunotherapy, LASSO, signature, tumor microenvironment

## Abstract

Breast cancer (BC) is one of the most common tumor types and has poor outcomes. In this study, a ubiquitination-related prognostic signature was constructed, and its association with immunotherapy response in BC was explored. A list of ubiquitination-related genes was obtained from the molecular signatures database, and a ubiquitination-related gene signature was obtained by least absolute shrinkage and selection operator Cox regression. The genes, *TCN1*, *DIRAS3*, and *IZUMO4*, had significant influence on BC outcomes. Patients were categorized into two clusters—a high-risk group with poor survival and a low-risk group with greater chances of controlling BC progression. Univariate and multivariate Cox regression analyses revealed that the risk signature was an independent prognostic factor for BC. Gene set enrichment analysis suggested that the high-risk group was enriched in cell cycle and DNA replication pathways. The risk score was positively linked to the tumor microenvironment and negatively correlated with the immunotherapy response. The IC50 values for rapamycin were higher in the low-risk group, whereas those for axitinib, AZD6244, erlotinib, GDC0941, GSK650394, GSK269962A, lapatinib, and PD0325901 were higher in the high-risk group. Therefore, the ubiquitination-related signature is considered a promising tool for predicting a BC patient’s immunotherapy response.

## Introduction

Breast cancer (BC) is the predominant type of cancer in women. The incidence rate of BC has risen steadily over the past decades, affecting nearly 100,000 patients worldwide annually (Britt et al., 2020; Sung et al., 2021). Currently, BC is commonly treated with chemotherapy either alone or in combination with other treatments. However, resistance to the available BC chemotherapeutic drugs is growing. The TNM staging system is widely used for assessing drug responsiveness, but it accounts for only a portion of the survival variation in BC patients (Edge et al., 2019). High-coverage and next-generation sequencing technologies are very efficient and sensitive, making it possible to compare whole-genome sequences of selected patients. Therefore, a panel of robust genetic biomarkers would be beneficial for improving the accuracy in estimating the prognosis of BC patients.

Ubiquitination, the addition of a ubiquitin molecule to the substrate, is an important function that involves a series of adaptive mechanisms among strongly invasive, proliferating cancer cells (Morrow et al., 2015). Ubiquitination regulates a large repertoire of cellular functions and requires the presence of an E3 ligase, which transfers ubiquitin (Ub) to substrates. The control system determining the specificity of the process is closely associated with carcinogenesis and tumor progression (Sun, 2006). Moreover, the ubiquitylation function may be directed towards degradation by the proteasome (degradative ubiquitylation) or towards altering the function (regulatory ubiquitylation) (Bergmann, 2010). Ubiquitination could be considered an essential hallmark of cancer; hence, it has been extensively investigated in previous studies using statistical tests and machine learning to integrate transcriptomics and ubiquitination into the underlying molecular mechanisms, with significant benefits (Popovic et al., 2014; Ge et al., 2018). Aberrant ubiquitination could underlie some of the heterogeneity of lung cancer, and drugs that target ubiquitin could offer effective new cancer treatments (Che et al., 2022). Thus, it is important to identify robust tumor-associated ubiquitination biomarkers, which could not only improve BC diagnosis and prognosis but also help develop novel therapeutic strategies.

To investigate the impact of genetic alteration of ubiquitination on BC, we explored the influence of ubiquitination-related genes (URGs) on BC, identified new prognostic groups, and constructed a predictive risk signature based on URG function.

## Methods

### Data profiles

Raw transcription statistics (fragments per kilobase of exon model per million reads mapped [FPKM] and count data) and corresponding clinical information (age, sex, pathological stage, TNM stage, survival time, and survival position) were downloaded from the Cancer Genome Atlas (TCGA, https://www.cancer.gov, BRCA project). The log2 transformation was used to normalize the TCGA-BRCA cohort. We downloaded two microarray datasets (GSE20685 and GSE25066) (Kao et al., 2011; Baldasici et al., 2022) from the Gene Expression Omnibus (GEO) database (https://www.ncbi.nlm.nih.gov/geo/) and used them to validate the cohorts. Batch effects among the TCGA-BRCA and GEO datasets were eliminated by using the “ComBat” method in the sva package from R. The BC models of Tang et al., Wu et al., and Hu et al. were used to test and demonstrate the advantages of our ubiquitination-related prognostic signature (Tang et al., 2020; Wu et al., 2020; Hu et al., 2022).

### Consensus clustering analysis of ubiquitination-related genes

Seventy-nine ubiquitination-related genes (URGs) were downloaded from the MSigDB database (http://www.broad.mit.edu/gsea/msigdb/), including gene symbol, official full name, ensembl ID, and gene type (Supplementary File S1). The R package ConsensusClusterPlus was used to conduct an agreement-unsupervised clustering analysis to sort BCs into distinct clusters according to the expression of URGs (Supplementary File S2) (Wilkerson and Hayes, 2010).

### Relationship between molecular subtypes, clinical features, and BC prognosis

To examine the medical value of the two subtypes identified by harmony gathering, we compared the relationships between the different molecular subtypes, clinicopathological characteristics, and prognoses. Patient characteristics included sex, age, and TNM stage. Differences in overall survival (OS) between different subtypes were also evaluated using Kaplan–Meier curves generated by the “survminer” and “survival” R packages (Rich et al., 2010).

### Tumor microenvironment of ubiquitination-related clusters

Immune cell infiltration was established using the MCPcounter algorithm to evaluate the tumor microenvironment (TME) of ubiquitination-related clusters (Becht et al., 2016). Differences in immune and stromal cell types were identified using the limma algorithm and presented with ‘violin’ plots (Ritchie et al., 2015). The data are shown using the R packages, limma and MCPcounter.

### Construction and authentication of a predictive signature for BC

We normalized raw mRNA expression data from the TCGA-BRCA project and, based on the differentially expressed genes (DEGs) among ubiquitination-related clusters, we identified the downstream genes influenced by URGs using the R limma package (*p* < 0.05 and |log_2_ FC| > 1 denoted statistical significance) (Ritchie et al., 2015). Univariate Cox regression analyses were conducted to classify prognostic DEGs (*p* < 0.05). Then, least absolute shrinkage and selection operator (LASSO) regression analysis was performed to classify the prognostic DEGs and build a predictive signature for BC (Tibshirani, 1997).

$$prognosis\;index\;\left(PI\right)=\sum_{i=1}^{n}Coef\left(i\right)*Expr\left(i\right).$$

BC patients from TCGA-BRCA were divided into low- and high-risk subgroups based on the median TCGA-BRCA risk score. The whole TCGA-BRCA dataset was set as a training subset, then half the TCGA-BRCA patients were arbitrarily selected as the examination subset. The R survival package was used to compare the survival between the two groups using the Kaplan–Meier plotter in the training and test subsections. In addition, receiver operating characteristic (ROC) curves with area under the curve (AUC) standards for 1-, 3-, and 5-year survival were used to evaluate the prognostic ability of the ubiquitination-related signature for BC (Hajian-Tilaki, 2013). The survival status of BC patients in the TCGA-BRCA dataset is presented. Principal component analysis (PCA) and *t*-distributed stochastic neighbor embedding (*t*-SNE) analysis were used to evaluate the separation of low- and high-risk BC (Ringnér, 2008; Belkina et al., 2019). To further address the prognostic attributes of the signature, univariate and multivariate Cox regression analyses were performed to determine the independent risk factors for BC, with covariates such as the risk score, age, and TNM stages.

### Establishment and verification of the nomogram

To improve the prognostic value of the signature, ubiquitination-related genes and new characteristics were used to build a nomogram with the “rms” and “regplot” R packages (Iasonos et al., 2008). The nomogram was used to predict the 1-, 3-, and 5-year survival rates of patients, and calibration curves were used to assess the accuracy of the nomogram (Austin et al., 2020). ROC and DCA analyses were performed to evaluate the stability of the prognostic nomogram (Van Calster et al., 2018).

### DEG identification and functional annotation

To explore the potential cellular functions and enriched pathways of genes downstream of URGs, functional enrichment analyses, including GO and KEGG, were performed on the DEGs using the “clusterprofiler” R package (Yu et al., 2012). Gene set enrichment analysis (GSEA) was conducted for differentially enriched pathways in low- and high-risk BC (Yu et al., 2012).

### Subgroup analysis of low- and high-risk BC

To determine the correlation between the ubiquitination-related signature and the clinical characteristics, risk differences between low- and high-risk BC were assessed in subgroups by age, sex, clinical stage, tumor grade, and TNM stage. In addition, the survival differences of low- and high-risk BC in different subgroups were analyzed using Kaplan–Meier survival analysis. The results are presented with significance set at *p* < 0.05.

### TME and immunotherapy response analysis of low- and high-risk BC

The correlation between ubiquitination-related signatures and immunoregulatory genes, immune checkpoint genes, and various types of immune cells was evaluated using Pearson’s coefficient. A heatmap of immune cell infiltration in high- and low-risk BC was generated using Timer, Xcell, Quantiseq, MCPcounter, EPIC, and Cibersort procedures (Becht et al., 2016; Aran et al., 2017; Li et al., 2017; Chen et al., 2018; Plattner et al., 2020; Racle and Gfeller, 2020). Expression of the immunotherapy targets CD47 and CTLA4 was determined, and TIDE and MSI scores were calculated to predict the immunotherapy response. Potential anti-PD-1 treatment efficiency was evaluated using the IMvigor 210 cohort (Balar et al., 2017).

### Drug sensitivity evaluation of low- and high-risk BC

The R package “pRRophetic” was used to predict drug sensitivity in high- and low-risk BC patients (Geeleher et al., 2014). The data were extracted from the GDSC database (https://www.cancerrxgene.org/), and the half-maximal inhibitory concentration (IC50) was used as the index.

### Cells and cell culture

Normal breast epithelial cells (MCF-10A) and cancer cells (BT-549) were acquired from the American Type Culture Collection (Manassas, VA, United States). The cells were incubated with RPMI-1640 medium (Gibco, Carlsbad, CA, United States), containing 10% fetal bovine serum (Gibco, Carlsbad, CA, United States) and 1% penicillin-streptomycin (Gibco, Carlsbad, CA, United States). All cells were cultured at 37°C with 5% CO_2_.

### RT-qPCR

Total RNA was extracted from MCF-10 A and BT-549 cells using TRIzol reagent (Invitrogen), and reverse-transcribed into cDNA using PrimeScript^®^ RT master mix (Perfect Real Time, Takara Bio, Shiga, Japan). RT-qPCR was performed using the qPCR master mix (Promega, Madison, WI, United States). Reactions were performed in triplicate, and Ct values were normalized to the endogenous housekeeping gene, *β-actin*, using the 2^−ΔΔCT^ method.

The primer sequences were as follows: 5′-GAT​TAC​CGC​GTC​GTG​GTA​GTC-3′ (forward) and 5′-TCA​ATG​GTC​GGC​AGG​TAC​TCA-3′ (reverse) for *DIRAS3*; 5′-GGA​GCG​ACG​ACA​CGA​TGA​AG-3′ (forward) and 5′-CAG​CTC​GTT​GGG​GAA​ATA​CCC-3′ (reverse) for *IZUMO4*; 5′-CCC​CTA​GTG​GGG​CTC​TTA​CT-3′ (forward) and 5′-CAG​AGG​TTT​TAG​GCG​GAT​GTA​G-3′ (reverse) for *TCN1*; and 5′-TGA​CGT​GGA​CAT​CCG​CAA​AG-3′ (forward) and 5′-CTG​GAA​GGT​GGA​CAG​CGA​GG-3′ (reverse) for *β-actin*.

### Statistical analysis

We used the R software (version 4.0.3) to perform all calculations and produce the statistical data. A *p* < 0.05 was considered statistically significant.

## Results

### Data

The expression ranks of 79 URGs, obtained from the Molecular Signatures Database (MSigDB) in the GSEA database, were measured in BC and normal breast samples from TCGA database using the Wilcoxon signed-rank test (Supplementary File S1).

### Identification of ubiquitination-related clusters in BC

**FIGURE 1:**
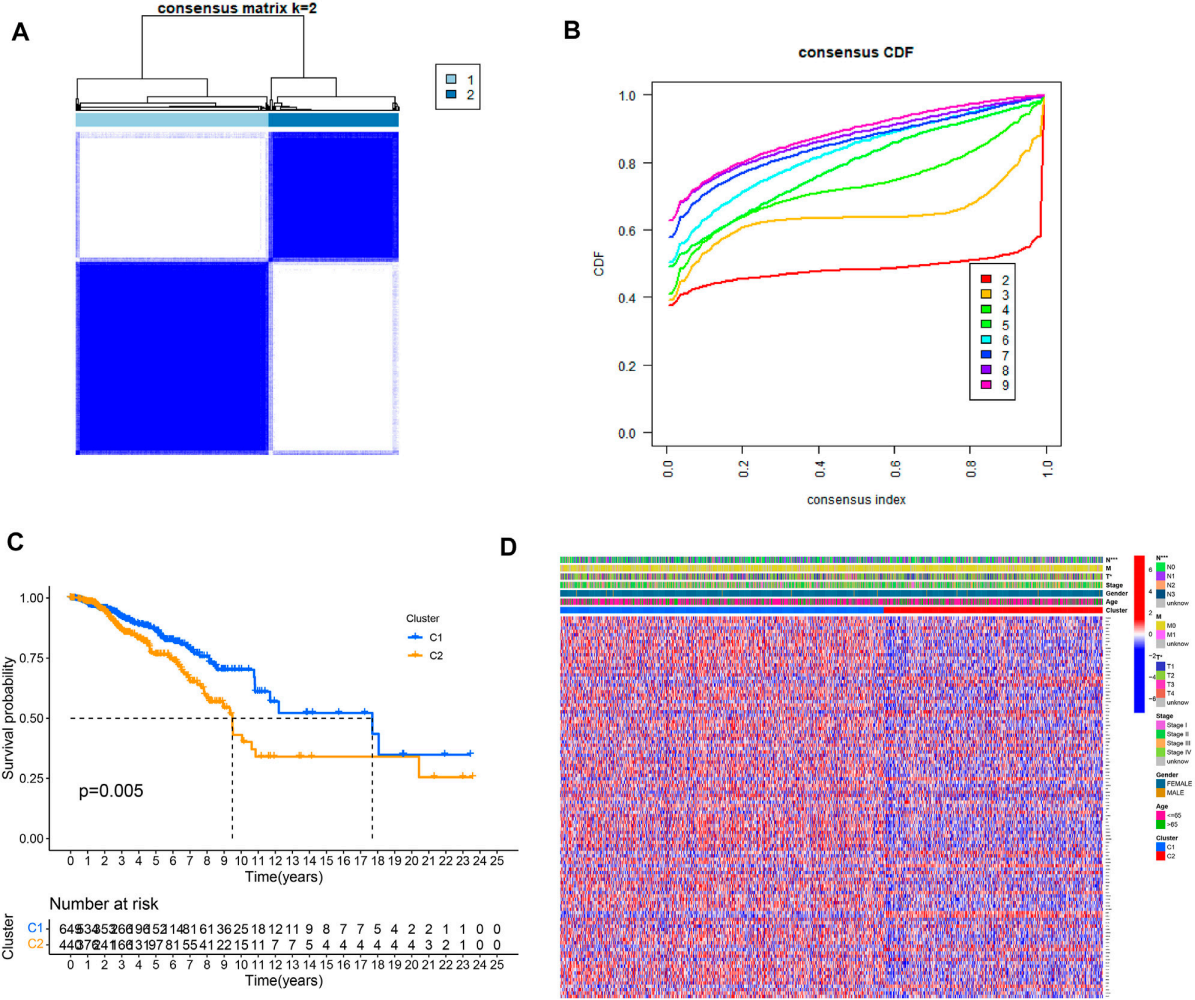
Clinical pathology and prognostic value of two distinct subtypes of patients divided by consistent clustering. **(A)** Consensus matrix heatmap defining two clusters (*k* = 2) and their correlation area. **(B)** Consensus CDF in consistent clustering (*k* = 2–9). **(C)** Survival curve showing overall survival between C1 and C2. **(D)** Differences in clinical pathology features between the two clusters. C1, cluster 1; C2, cluster 2.

**FIGURE 2:**
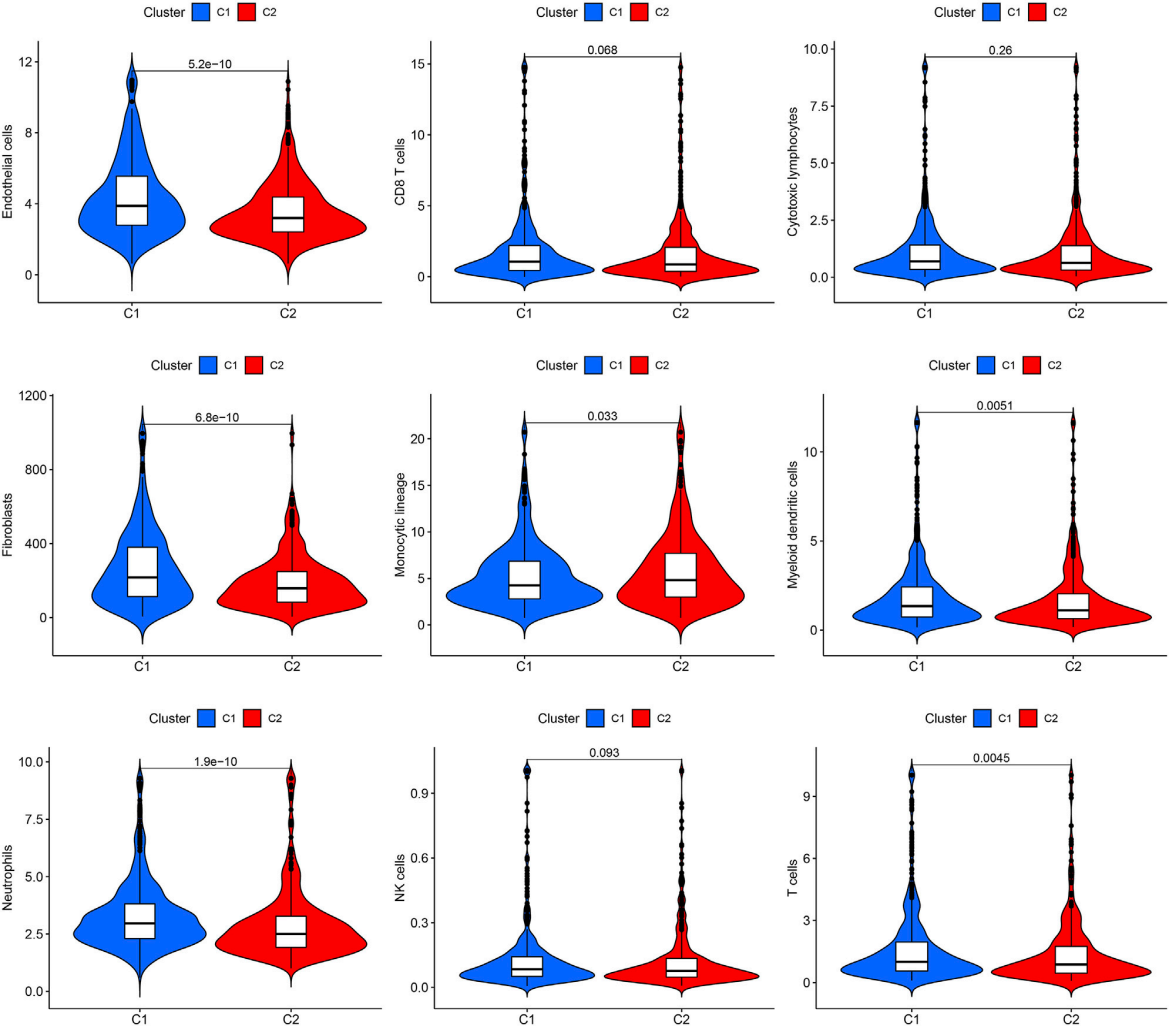
Immune cell infiltration in the two clusters. Violin plot of endothelial cells, CD8^+^ T cells, cytotoxic lymphocytes, fibroblasts, monocyte lineage, myeloid dendritic cells, neutrophils, NK cells, and T cells in the two clusters. C1, cluster 1; C2, cluster 2.

To understand how URGs are involved in tumorigenesis, we used an agreement clustering algorithm to classify BC based on the mRNA expression of 79 URGs (Supplementary File S2). In our dataset, *k* = 2 was considered the best choice for organizing the cohort into clusters 1 and 2 (Figure 1A). The consensus CDF of consistent clustering (*k* = 2-9) is shown in Figure 1B. The Kaplan–Meier curve revealed longer survival in cluster 1 BC than in cluster 2 BC (*p* = 0.005; Figure 1C). In addition, the clinicopathological topographies of the different BC subtypes exhibited important differences in URG expression and clinicopathological characteristics (Figure 1D). As shown in Figure 1D, cluster 1 was preferentially related to a lower N (*p* < 0.001) and T stage (*p* < 0.05) than cluster 2. The MCPcounter study showed that immune cell infiltration, including T-cell neutrophils, monocytic lineage cells, myeloid dendritic cells, fibroblasts, and endothelial cells, was significantly enriched in cluster 1 compared to that in cluster 2 (Figure 2).

### Identification of gene clusters using DEGs

**FIGURE 3:**
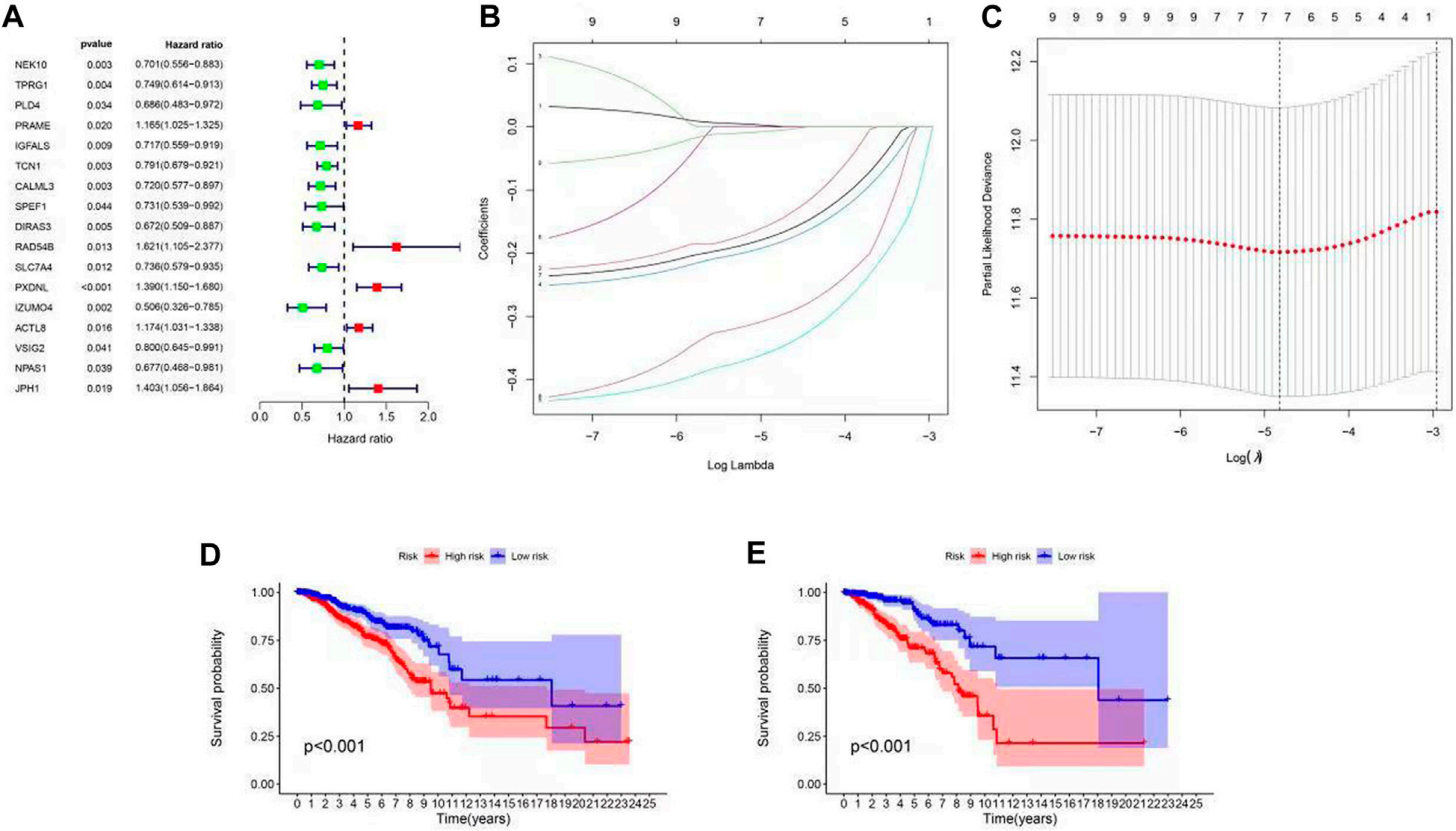
Construction of ubiquitination-related signatures from different clusters in patients with BC. **(A)** Forest plot of 17 differentially expressed genes between C1 and C2 identified as prognosis-related using univariate Cox analysis. **(B,C)** LASSO regression analysis and partial likelihood deviance of nine differentially expressed genes between C1 and C2. **(D,E)** Survival curve of high- and low-risk groups (*p* < 0.001). **(D)** Training set, **(E)** test set.

To determine the biological function of a ubiquitination pattern, we identified ubiquitination subtype-connected DEGs using the R package, limma. We examined the prognostic value of DEGs through BC progression using univariate Cox regression analysis to determine the potential relationship between the gene expression levels of the patient and two clusters in terms of survival. The results confirmed that 17 DEGs were associated with survival (*p* < 0.01) (Figure 3A). Thus, *PRAME*, *RAD54B*, *PXDNL*, *ACTL8*, and *JPH1* were considered high-risk genes (HR > 1), whereas *NEK10*, *TPRG1*, *PLD4*, *IGFALS*, *TCN1*, *CALML3*, *SPEF1*, *DIRAS3*, *SLC7A4*, *IZUMO4*, *VSIG2*, and *NPAS1* were considered protective genes (HR < 1). We then used LASSO Cox regression to classify the best risk score model for predicting survival in patients with BC (Figures 3B, C). The median risk score of patients with BC was identified as the threshold. As the training subset, all the patients with BC in TCGA-BRCA were divided into high- and low-risk groups. There was a significant difference in OS between the high- and low-risk groups according to the Kaplan–Meier curve (*p* < 0.001) (Figure 3D). Similarly, for the test set, the randomly selected patients (half of the total) with BC in TCGA-BRCA were divided into high- and low-risk groups according to the median risk score. Consistent with the OS in the training set, a high survival probability was observed in the low-risk group in the test set (Figure 3E).

### ROC curve analysis of the BC prognosis risk scoring model

**FIGURE 4:**
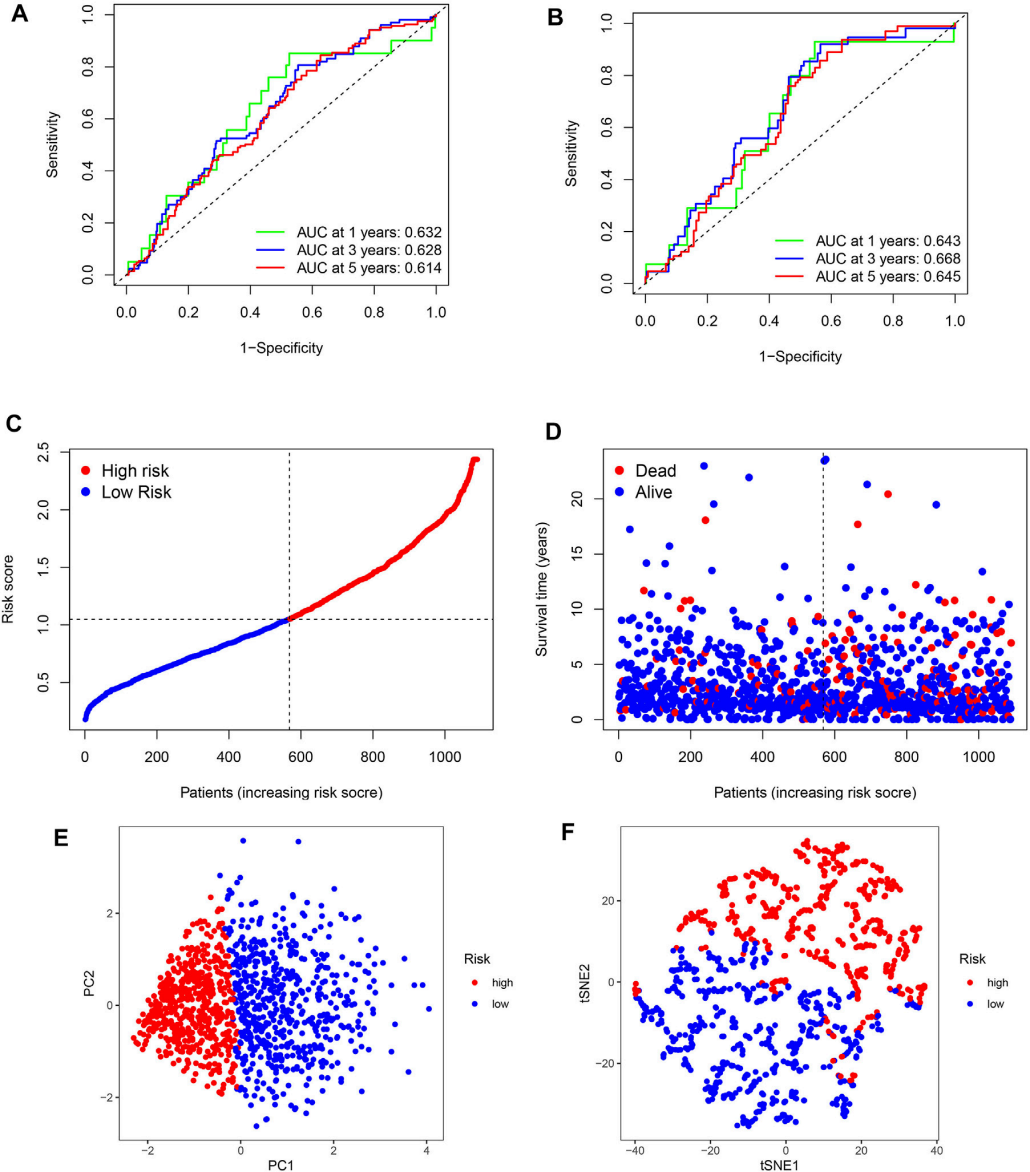
ROC curves and risk scores of the signature in predicting OS of patients with BC. **(A,B)** 1-, 3-, and 5-year ROC curves to evaluate the accuracy of our model. **(C,D)** Distribution of risk scores and patient living status. **(E,F)** PCA and *t*-SNE analysis based on the prognostic model. High- and low-risk patients are represented by red and blue dots, respectively, according to the URG score.

To determine whether the BC signature was predictive, ROC curves were generated. ROC curve analysis revealed a significant prognostic effect for BC based on the risk model group, the AUC values for 1-, 3-, and 5-year survival being 0.632, 0.628, and 0.614 in the training set and 0.643, 0.668, and 0.645 in the test set, respectively (Figures 4A, B). Our survival and risk status maps showed that the high-risk groups had more deaths than the low-risk groups (Figures 4C,D). In addition, an important difference was observed between the two clusters in terms of ubiquitination-related transcription profiles through PCA and *t*-SNE analysis (Figures 4E,F).

### Independent validation of the signature’s stability and advantages

To further corroborate the advantages and stability of our ubiquitination-related signature, three breast cancer models were selected for comparison. The models of Tang et al., Wu et al., and Hu et al. showed a lower C-index than our signature (0.509, 0.598, and 0.645-0.646) (Supplementary Figure S1). Consequently, we determined the K-M survival and performed ROC analyses on the GSE20685 and GSE25066 datasets. The results confirmed that the low- and high-risk groups had a significantly different prognosis in GSE20685 and GSE25066 (*p* = 0.006; *p* = 0.037). The two ROC analyses showed acceptable stability for 1-, 3-, and 5-year OS (0.687, 0.647, and 0.636; 0.641, 0.656, and 0.632) (Supplementary Figure S1).

### Structure and validation of the analytical nomogram

**FIGURE 5:**
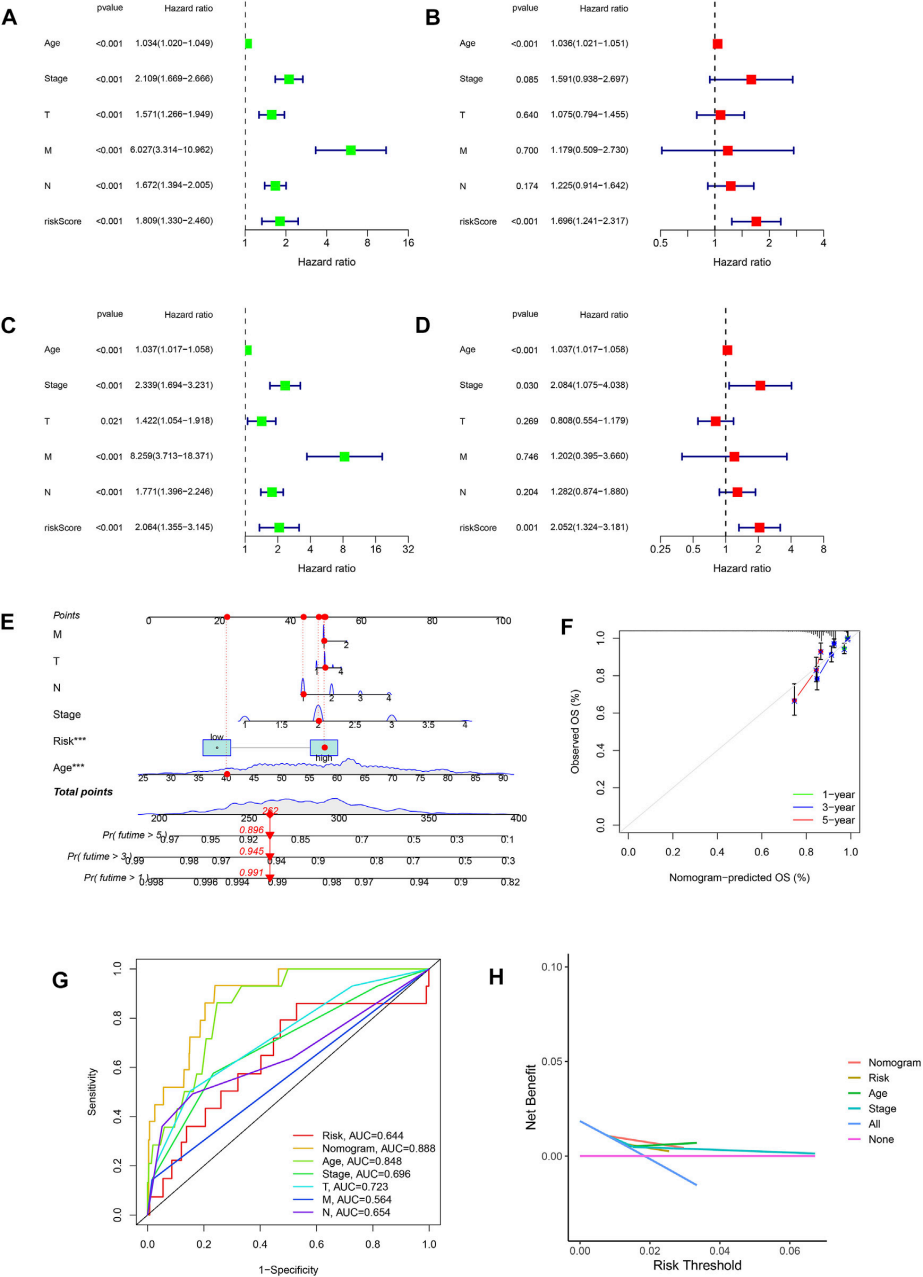
Relationship between risk scores and clinical features. **(A,B)** Univariate and multivariate Cox analyses considering risk score, age, tumor stage, and TNM stage in the training cohort. **(C,D)** Univariate and multivariate Cox analyses considering risk score, age, tumor stage, and TNM stage in the test cohort. **(E)** Nomogram combining risk score and clinicopathological factors. **(F)** Calibration plots established to compare the proposed nomogram with an ideal model. **(G)** AUCs showing that this nomogram had higher accuracy in OS than other factors. **(H)** Net benefit of the proposed nomogram, risk group, age, and tumor stage.

The risk scores were identified as independent analytical indicators using multivariate and univariate Cox regression analyses (Figures 5A–D). Based on several clinical parameters and risk scores, separate numerical probabilities for OS were generated using a prognostic nomogram (Figure 5E). Through calibration curves, the nomogram predicted 1-, 3-, and 5-year OS well compared to the ideal model (Figure 5F). ROC curves were used to evaluate the prognostic accuracy of the nomogram and the other signatures. A high prognostic value was indicated by an area under the ROC curve of 0.888 (Figure 5G). Compared to risk, age, and stage, the nomogram produced a greater net benefit in forecasting OS according to DCA (Figure 5H). Furthermore, the nomogram demonstrated a better net benefit than age and tumor stage, indicating its reliability and predictability.

### Clinical utility and functional enrichment analysis of the predictive signature

**FIGURE 6:**
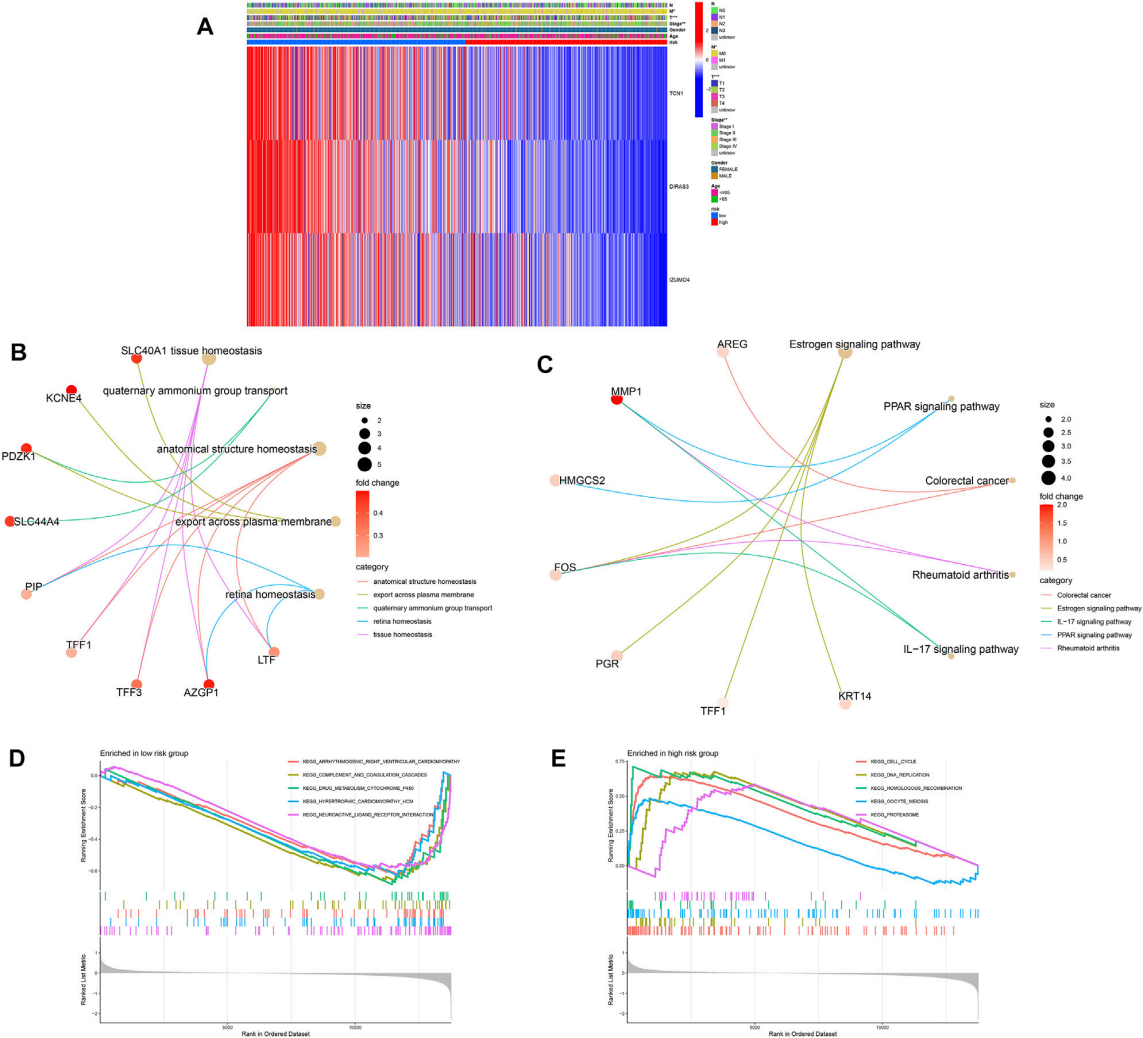
Relationship between clinical features and gene expression in high- and low-risk groups. **(A)** Heatmap of the two clusters along with clinicopathological characteristics and differentially expressed genes. **(B,C)** GO and KEGG analyses of differentially expressed genes between high- and low-risk groups. **(D,E)** Gene set enrichment analysis in the low-risk **(D)** and high-risk **(E)** groups.

We also examined the association between ubiquitination-related predictive factors and medical variables. A heatmap was constructed to display the expression levels of the three signature URGs in the high- and low-risk groups (Figure 6A). The low-risk group had high expression levels of *TCN1*, *DIRAS3*, and *IZUMO4*. Genes associated with ubiquitination-related prognostic signatures were significantly enriched in biological processes related to homeostasis (Figure 6B). KEGG analysis revealed pathways related to immune function and cancer (Figure 6C), suggesting that ubiquitination plays a pivotal role in the immune regulation of the TME. To further explore this mechanism, GSEA plots showed the presence of metabolic, cell cycle, cell communication, and DNA replication pathways in the high- and low-risk groups (Figures 6D, E).

**FIGURE 7:**
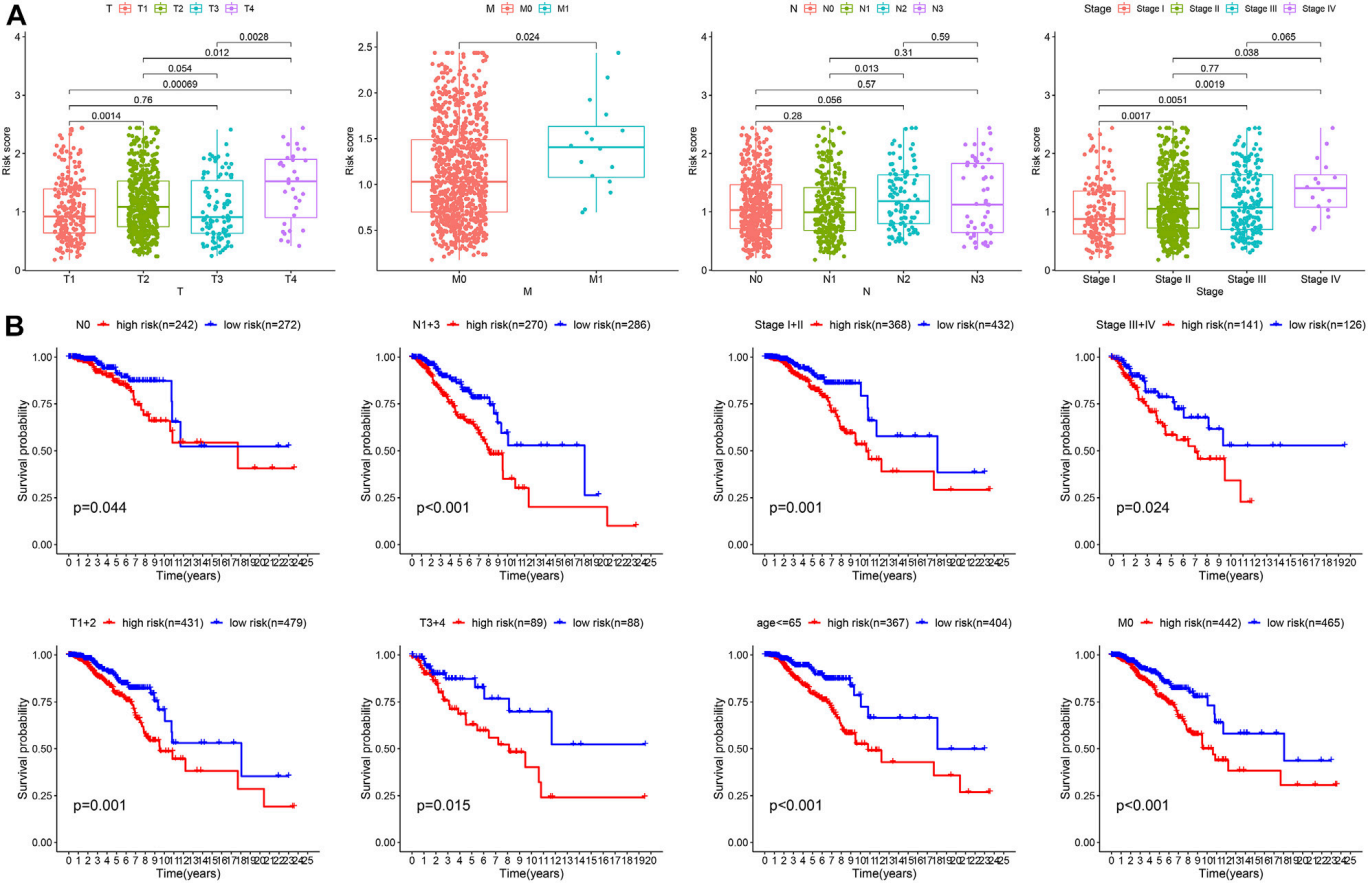
Clinical correlation and stratification analyses of the signature. **(A)** Correlation between the risk score and TNM and tumor stages. **(B)** Stratified survival analysis between high- and low-risk groups.

The function of the important risk scores in disease progression was assessed by evaluating the relationship between the risk score in the predictive signature and clinicopathological features. High-risk scores were associated with advanced M1 stages, T stages, and high-grade tumors, suggesting a strong correlation between risk scores and low prognosis in BC. High-risk scores were seen in the most advanced clinicopathological stages, T4, stage IV, and M1 (Figure 7A). Next, we performed survival examinations stratified by age, TNM stage, and clinical stage to better estimate the survival consequences and determine the broad applicability of the predictive signature (Figure 7B). Patients in the high-risk group had significantly shorter OS than those in the low-risk group for cases with N0 (*p* = 0.044), N1+3 (*p* = 0.001), clinical stages I and II (*p* = 0.001), clinical stages III and IV (*p* = 0.024), T1+2 (*p* = 0.001), T3+4 (*p* = 0.015), age ≤65 (*p* < 0.001), and M0 (*p* < 0.001) (Figure 7B).

### Calculation of TME and checkpoints between the high- and low-risk groups

**FIGURE 8:**
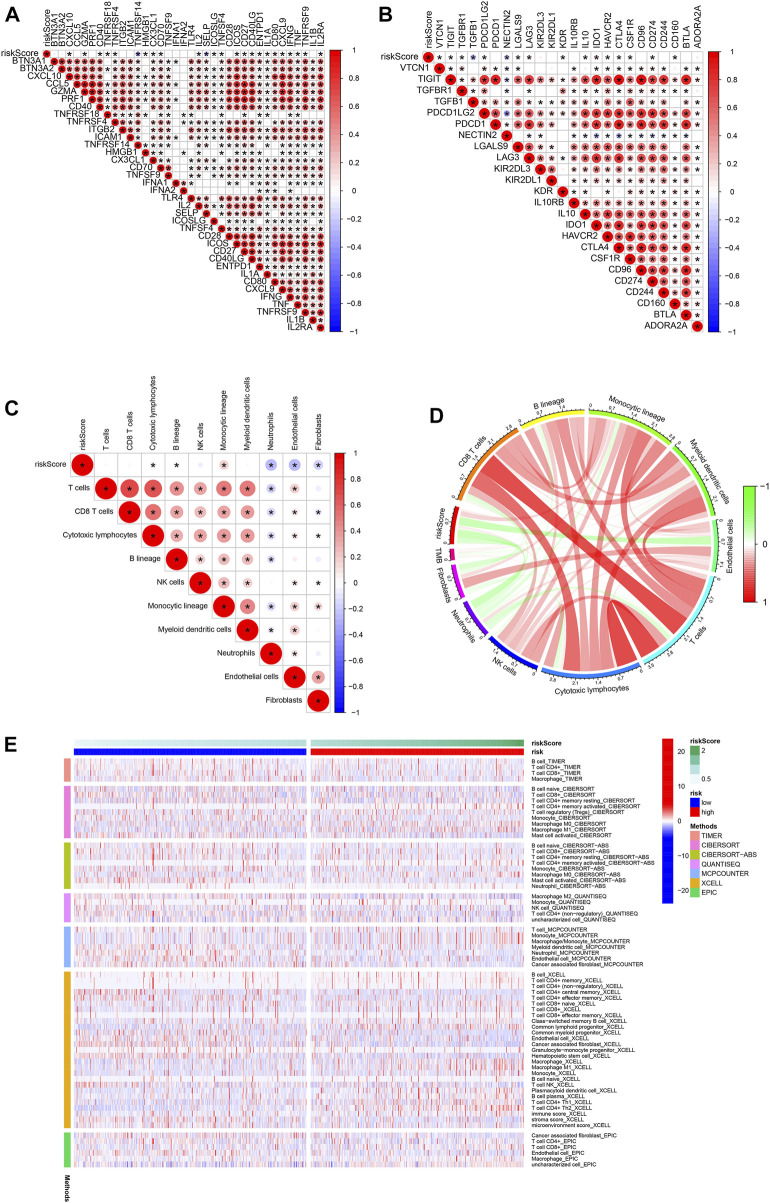
Association of immune signatures and immune cell infiltration with risk score. **(A,B)** Correlation of immune signatures and risk score. **(C,D)** Correlation of immune cell infiltration and risk score. **(E)** Clustering pattern of immune cell type in the high- and low-risk groups.

**FIGURE 9:**
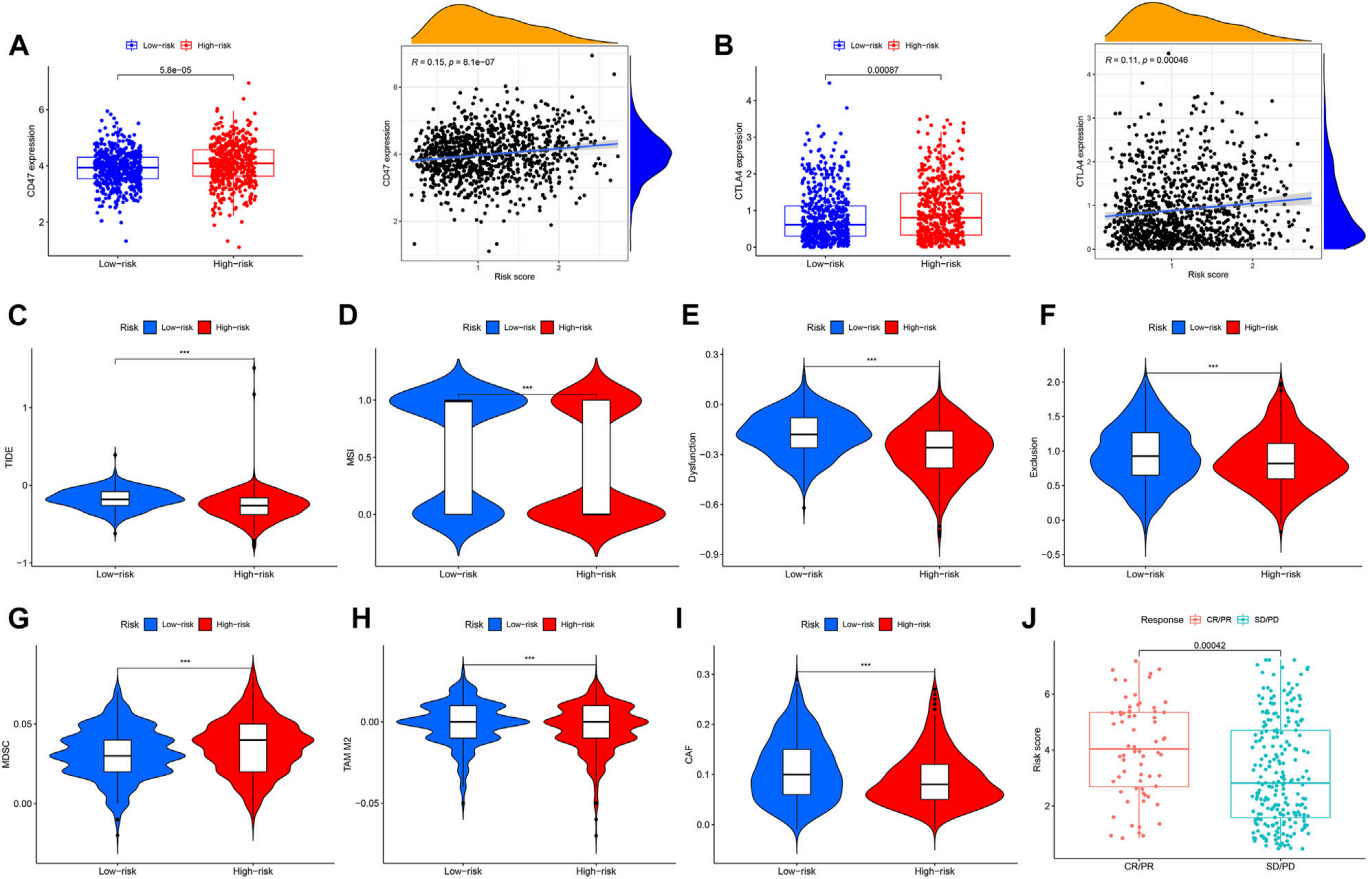
Correlation analysis and differential expression of immune checkpoints and TME scores in the two groups. **(A)** Bar plot of CD47 expression in the high- and low-risk groups and correlation of risk score and CD47 expression. **(B)** Bar plot of CTLA4 expression in the high- and low-risk groups and correlation of risk score and CTLA4 expression. **(C–I)** Values of TIDE, MSI, Dysfunction, Exclusion, MDC, TAM M2, and CAF in the high- and low-risk groups. **(J)** Comparison of the risk score in the CR/PR and SD/PD groups. CR, complete response; PR, partial response; SD, stable disease; PD, progressive disease, ***p < 0.001.

We assessed the correlation between risk scores and the abundance of immune cells. The association of biomarkers of risk scores and immune cells is illustrated in Figures 8A, B Forty immune checkpoint genes showed a significant correlation with risk score. As shown in Figures 8C, D, the risk scores were positively correlated with the monocytic lineage and negatively correlated with cytotoxic lymphocytes, the B lineage, neutrophils, endothelial cells, and fibroblasts. The association between the proposed model and the numbers of immune cells was investigated using the Xcell, Cibersort, Cibersort-ABS, EPIC, MCPcounter, TIMER, and QuantiSeq algorithms, which showed similar results (Figure 8E). CD47 and CTLA4 were highly expressed in the high-risk group compared with the low-risk group, implying that CD47 and CTLA4 expression strongly correlated with higher risk (Figures 9A, B). A low-risk score was also associated with high TIDE, dysfunction, exclusion, TAM M2, and CAF scores, whereas a high-risk score was associated with a high MDSC score (Figures 9C–I). Groups with low- and high-risk samples were significantly correlated with immunotherapy responses; high-risk BC patients showed better therapy responses (Figure 9J).

### Drug sensitivity analysis of high- and low-risk groups

**FIGURE 10:**
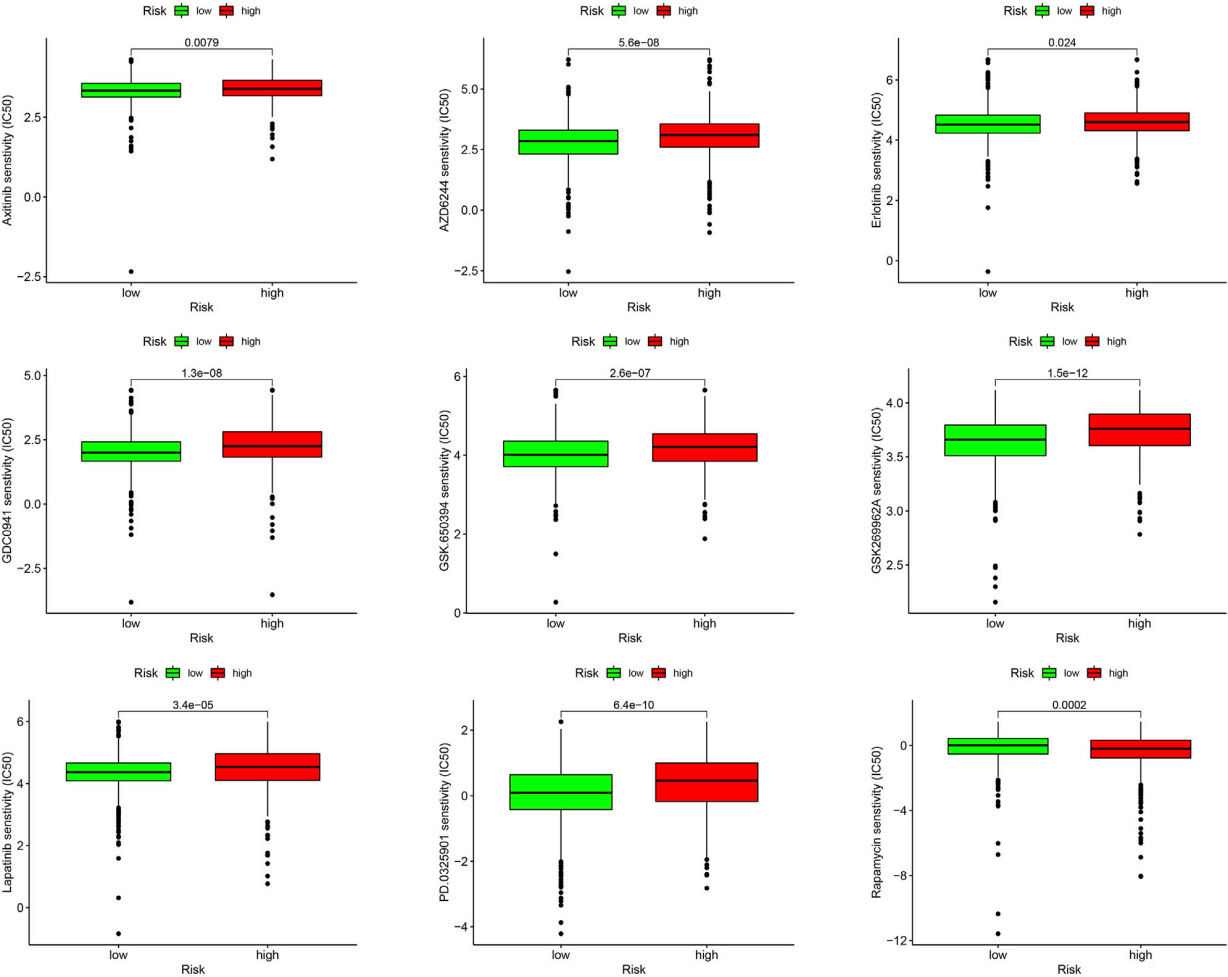
Drug sensitivity analysis between high- and low-risk groups.

We evaluated the sensitivities of patients in the low- and high-risk groups to chemotherapy drugs currently used to treat BC. Rapamycin had a low IC50 value in patients in the high URG score group, whereas axitinib, AZD6244, erlotinib, GDC0941, GSK.650394, GSK269962A, lapatinib, and PD0325901 had low IC50 values in patients in the low URG score group. The results confirmed the validity of the ubiquitination-related signature in predicting drug sensitivity (Figure 10).

### RT-qPCR

**FIGURE 11:**
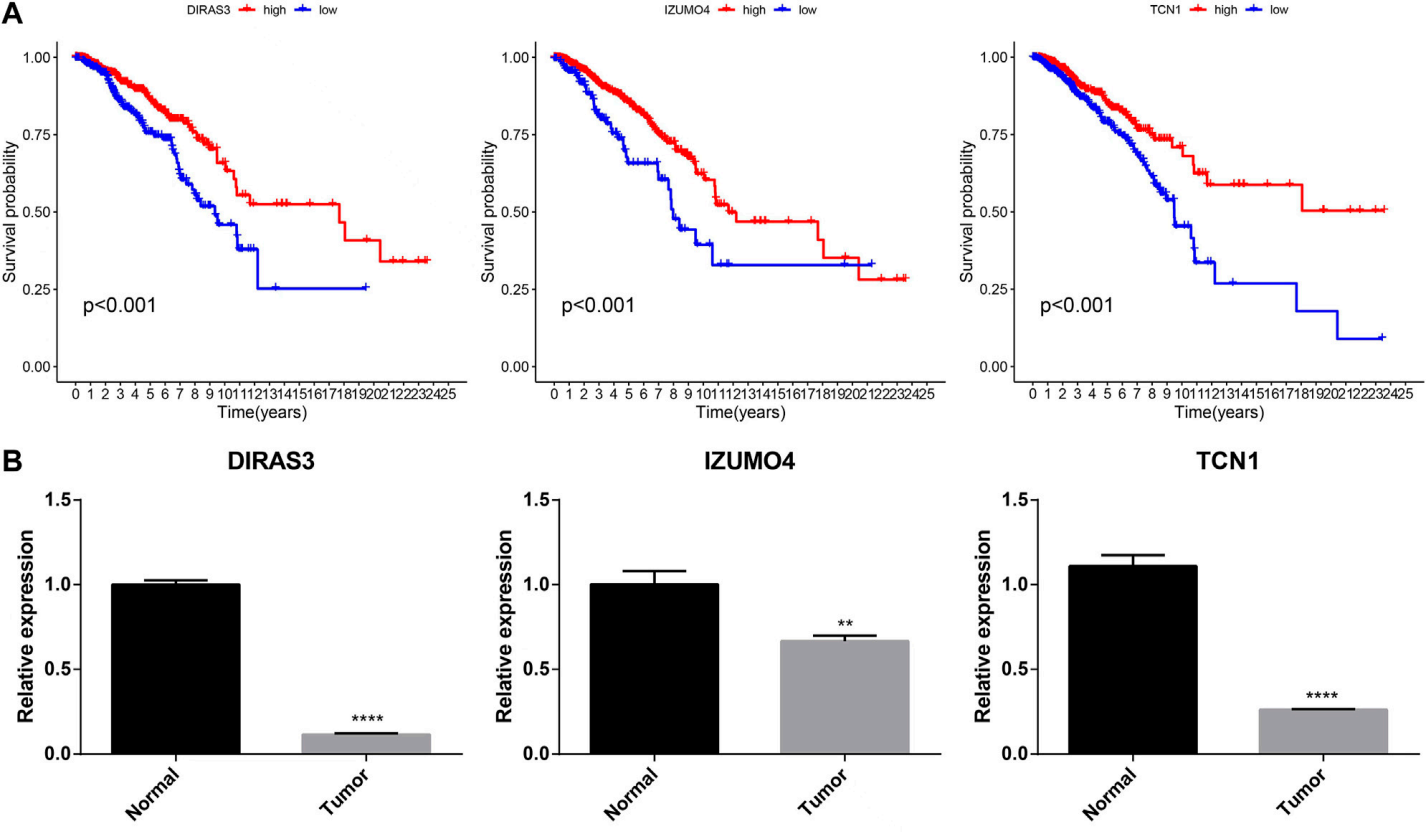
Prognostic and RT-qPCR validation of *DIRAS3*, *IZUMO4*, and *TCN1*. **(A)** Kaplan–Meier survival analysis for *DIRAS3*, *IZUMO4*, and *TCN1* in BC. **(B)** RT-qPCR of *DIRAS3*, *IZUMO4*, and *TCN1* in normal breast epithelial and BC cells.

Three genes that constituted the ubiquitination-related signature were confirmed as prognostic biomarkers for BC (Figure 11A). Low expression of *DIRAS3*, *IZUMO4*, and *TCN1* correlated with poor BC prognosis. RT-qPCR was used to measure transcription levels and compare the mRNA expression of *DIRAS3*, *IZUMO4*, and *TCN1* in BC. The results demonstrated that *DIRAS3*, *IZUMO4*, and *TCN1* had a lower expression in tumor cells than in normal breast epithelial cells (Figure 11B). The present analysis shows that *DIRAS3*, *IZUMO4*, and *TCN1* are suppressor oncogenes.

## Discussion

BC is characterized by high heterogeneity, which may affect its prognosis (Roulot et al., 2016). Early and small BC with complicated clinical features makes diagnosis and treatment difficult (Unger-Saldaña, 2014). At present, the major challenges in treating BC are primary diagnosis, accurate forecasting of tumor progression, and current treatment. Therefore, it is essential to examine the biological features of the disease and identify specific biomarkers that could enhance the accuracy of prognosis prediction, inform treatment personalization, and improve survival rates. With the development of next-generation sequencing technology, we can effectively study the related genetic characteristics and determine their effect on risk (Behjati and Tarpey, 2013). This will facilitate selection of the optimal treatment for individual patients and help them achieve their treatment goals. To improve the outcomes of patients with BC, it will be necessary to conduct clinical trials to evaluate the benefits of incorporation of preclinical results and application of molecular-guided treatment. URGs are regulators of tumor cell cycle, gene expression, and progression (Deng et al., 2020). However, there is little understanding of the role of protein ubiquitination in the BC microenvironment. Thus, URG in the TME needs to be studied further.

Ubiquitination is a post-translational modification that plays a major role in pathological and physiological processes (Dougherty et al., 2020). Abnormally expressed genes have been investigated in several studies. *DERL1* acts as a vital regulator via interaction with *UBE2C* during oncogenic activities in BC (Zeng et al., 2020). One study uncovered pivotal functions of Derlin-1 in the regulation of ER stress-induced preventive control and ubiquitination by the HRD1 E3 ubiquitin ligase prior to deprivation, suggesting an important role of *DERL1* in homeostasis (Kadowaki et al., 2018). The present study showed that *DERL1* was significantly upregulated in BC and corresponded with a poor prognosis. Furthermore, we found that *UBA1*, *UBE2A*, *UBE2C*, *UBE2G1*, *UBE2T*, and *WAC* were significantly positively associated with poor BC prognosis.

In our study, two ubiquitination clusters were constructed and verified to improve BC outcome prediction. Cluster 1 was associated with better BC prediction than cluster 2. Higher N and T stages were observed in cluster 2 than in cluster 1, indicating that the cluster 2 group may be associated with a higher tumor stage of BC. Comparing immune cell infiltration in the two clusters, we found that cluster 1 had the most infiltration of endothelial cells, T cells, neutrophils, and myeloid dendritic cells. The robust ubiquitination-related gene signature was then verified by LASSO Cox regression analysis. Patients with a low-risk score had better OS than those with high-risk scores; 1-, 3-, and 5-year ROC curves confirmed the positive performance of the signature. Additional stratified existence analyses of various medical subgroups also established the signature’s robust predictive power. Multivariate and univariate Cox analyses revealed that the signature had independent predictive value. Using a nomogram model, we improved the efficacy of the analytical signature by considering age, risk scores, tumor stage, and TNM. This important model showed excellent accuracy in predicting the survival rates of BC patients. There were significant differences in immunocyte penetration and immunologic function between the low- and high-risk groups, and TME scores, immune checkpoints, and drug susceptibilities differed significantly between the two groups.

In another experiment, we identified three specific genes associated with the prognostic signature: *TCN1*, *DIRAS3*, and *IZUMO4*. A prognostic model for lung adenocarcinoma based on eight genes (*TCN1*, *COL1A1*, *SPOK2*, *PCP4*, *S100P*, *CAV2*, *GPX3*, and *ASPM*) has been previously constructed (Tu et al., 2021). Additionally, bioinformatics analyses have confirmed that the mRNA expression of *TCN1* was upregulated in common colon cancer (Liu et al., 2020); both Yang’s study and our model identified this gene. The expression of *TCN1* was significantly higher in the low-risk/high-survival group, than in the high-risk group. Low *TCN1* expression was associated with malignancy in BC, suggesting that it should be further evaluated for its specific predictive value. The expression levels of two other genes, *DIRAS3* and *IZUMO4*, were significantly higher in the low-risk group than in the high-risk group and were also useful in prognosis.

There are diverse models for breast cancer, based on metabolic heterogeneity, stromal immune phenotype, diverse cell-death patterns, cell necroptosis, plasmalogen deficiency, overactive fatty acid elongation biomarkers, and 4-mRNA metastasis-related genes (Xie et al., 2018; Zheng et al., 2020; Tomida et al., 2021; Yu et al., 2021; Xie et al., 2022; Zou et al., 2022). Yu et al. demonstrated that energy-related metabolic features in BC were related to glycolytic activity and survival. The clustering reflected intertumoral metabolic heterogeneity and could be used to personalize therapeutic strategies (Yu et al., 2021). In another study, 237 patients with triple-negative breast cancer (TNBC) from real-world cases and 533 patients with TNBC from public datasets were used to determine a stromal immune phenotype for TNBC. According to the density of stromal CD4^+^ T cells, γδ T cells, monocytes, M1 macrophages, and M2 macrophages, TNBC patients were divided into immune phenotypes, A and B, with different immune activities and prognosis (Zheng et al., 2020). A BC gene signature based on 27 metastasis-associated DEGs was also identified. Its prognostic ability was demonstrated and a nomogram was constructed based on mRNA signature, T stage, and N stage (Xie et al., 2018). Our signature revealed the heterogeneity of BC from the standpoint of ubiquitination, and we confirmed that it could be used as a tool to predict survival, immune activity, and therapy response.

A previous study acknowledged a risk model group of eight URGs that forecast risk for glioma (Wang et al., 2021). Several tumor-related signaling pathways were augmented in the high-risk group. TFs were also predicted to adjust the risk model for CD4 T cells and B cells; URGs and neutrophils were related to the risk model. In another study, FBXL6 played an important role in promoting hepatocellular carcinoma owing to the ubiquitination and stabilization of HSP90AA1, which contributed to tumorigenesis in hepatocellular carcinoma (Shi et al., 2020). Various enzymes are involved in ubiquitination, which can lead to cancer development; however, the underlying mechanisms need to be researched further (Wang et al., 2022). Ubiquitination is reversible, and de-ubiquitination can be used in common tumor treatment. In a study on BC, the E3 ubiquitin ligase, CHFR, was used to create double-strand breaks (DSBs) through poly(ADP-ribose) or PAR. Additionally, ALC1 (amplification in liver cancer-1) plays an important role in metastasis and invasion of BC, and poly(ADP-ribosyl)ated PARP1 stimulated ALC1 at DNA damage sites, emphasizing that the PBZ sphere of CHFR and PMD and Macro spheres of ALC1 are essential for the PAR interface. The common ubiquitination of ALC1 through CHFR is dependent on PARylation and directed to the degradation of PARylated ALC1 (Wang et al., 2019).

Several cancers, including bladder cancer and glioma, exhibited immune cell infiltration associated with URGs, which can provide novel therapeutic targets. The common ubiquitination-related subtypes showed significant differences in immune cell infiltration, stromal scores, ESTIMATE scores, and immune scores (Cai et al., 2021). According to the investigation of the immune microenvironment in gliomas, this risk grouping could be used as a guide for common glioma immunotherapy. An association study between risk ratings and immune cells showed that many immune cells were closely associated with risk scores. Lastly, high-risk BC patients showed higher expression levels of immune checkpoint genes than low-risk BC patients (Tang et al., 2022).

Six types of immune cells, including endothelial cells, fibroblasts, cytotoxic lymphocytes, neutrophils, monocytic cells, and B cells, were differentially expressed in different risk groups. Among these cells, monocytic lineages, which had a low survival rate, were significantly more abundant in the high-risk group than in the low-risk group. There were differences in MSI score, TIDE score, and immune checkpoint expression between the high- and low-risk groups. High-risk BC was significantly associated with low immune cell infiltration, which could support the discovery of new therapeutic methods. Despite recent advances in tumor immunotherapy, BC has not achieved satisfactory therapeutic benefits, and the mechanism underlying chemoresistance remains unclear; therefore, the development of multimodal therapies and bio-integration targets is necessary. The association between URG and BC requires further investigation.

In summary, our study identified a three-URG signature associated with immune infiltration and drug sensitivity. We confirmed the analytical model using training and test sets. The results showed that this signature could be used as a novel biomarker in many fields and provide a personalized treatment strategy for BC. The mechanism underlying URGs is still unknown, however, and needs to be explored in future studies.

## Data Availability

The original contributions presented in this study are included in the article/Supplementary Material. Further inquiries can be directed to the corresponding authors.

## Abbreviations

AUC, area under the curve; BC, breast cancer; DEG, differentially expressed genes; GSEA, gene set enrichment analysis; OS, overall survival; PCA, principal component analysis; ROC, receiver operating characteristic; TME, tumor microenvironment; *t*-SNE, *t*-distributed stochastic neighbor embedding; URG, ubiquitination-related gene.
