# Corrigendum: Prognostic implication and immunotherapy response prediction of a ubiquitination-related gene signature in breast cancer

**DOI:** 10.3389/fgene.2023.1217912

**Published:** 2023-05-23

**Authors:** Yangyang Guo, Qiaoqiao Chen, Yingjue Zhang, Xu Cheng, Kenan Cen, Ying Dai, Yifeng Mai, Kai Hong

**Affiliations:** ^1^ Department of Thyroid and Breast Surgery, Ningbo First Hospital, Ningbo, China; ^2^ Department of Thyroid and Breast Surgery, Ningbo Hospital of Zhejiang University, Ningbo, China; ^3^ Reproductive Medicine Center, The Affiliated Drum Tower Hospital of Nanjing University Medical School, Nanjing, China; ^4^ Key Laboratory of Reproductive Dysfunction Management of Zhejiang Province Assisted Reproduction Unit, Department of Obstetrics and Gynecology, Sir Run Run Shaw Hospital, Zhejiang University School of Medicine, Hangzhou, China; ^5^ Department of Molecular Pathology, Division of Health Sciences, Graduate School of Medicine, Osaka University, Suita, Japan; ^6^ Taizhou Hospital of Zhejiang Province Affiliated to Wenzhou Medical University, Taizhou, China; ^7^ The Affiliated Hospital of Medical School of Ningbo University, Ningbo, China

**Keywords:** breast cancer, ubiquitination, immunotherapy, LASSO, signature, tumor microenvironment

In the published article, there was an error in the **Author** list. The error **Author** list is “Kai Hong ^1,2†^, Qiaoqiao Chen ^3,4†^, Yingjue Zhang ^5†^, Xu Cheng ^6†^, Kenan Cen ^7^, Ying Dai^7^, Yifeng Mai^7*^ and Yangyang Guo^1,2^*.” The corrected author list appears below.

“Yangyang Guo ^1,2†^, Qiaoqiao Chen ^3,4†^, Yingjue Zhang ^5†^, Xu Cheng ^6†^, Kenan Cen ^7^, Ying Dai^7^, Yifeng Mai^7*^ and Kai Hong^1,2*^.”

The authors apologize for this error and state that this does not change the scientific conclusions of the article in any way. The original article has been updated.

