# Supplementary figures and images for: Prognostic implication and immunotherapy response prediction of a ubiquitination-related gene signature in breast cancer
Source: Front Genet. 2023 Jan 4;13:1038207. doi: 10.3389/fgene.2022.1038207 (PMC9845272; doi:10.3389/fgene.2022.1038207)

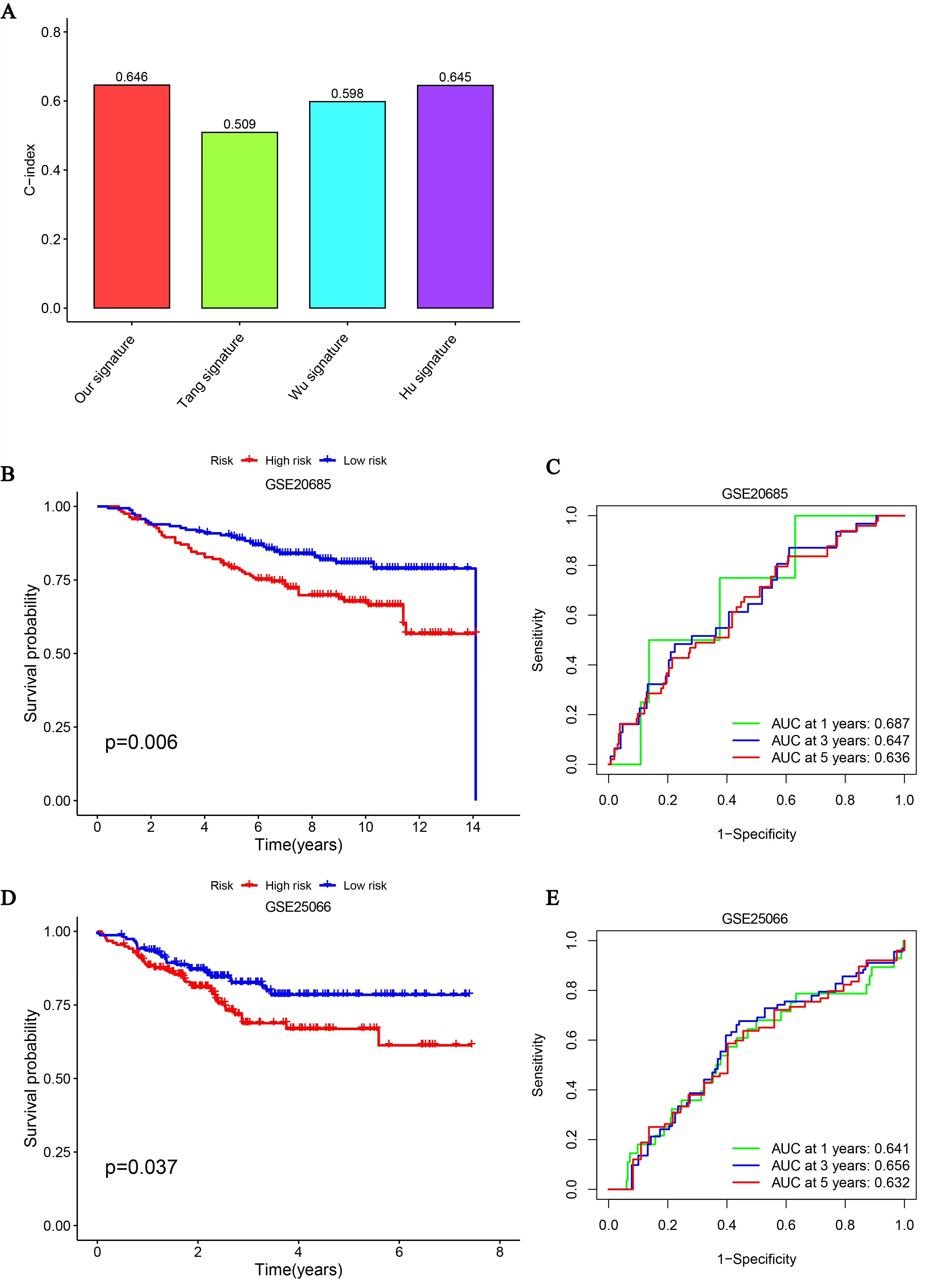

Supplement: Supplementary file 2 [file Image1.JPEG]
