# Supplementary material for: Prognostic implication and immunotherapy response prediction of a ubiquitination-related gene signature in breast cancer
Source: Front Genet. 2023 Jan 4;13:1038207. doi: 10.3389/fgene.2022.1038207 (PMC9845272; doi:10.3389/fgene.2022.1038207)
Supplement: Supplementary file 3 [file Table2.DOCX]

**Supplementary Table 2.** Expression data of ubiquitination-related genes across TCGA-BRCA

| Genes | p.value | HR | Low 95%CI | High 95%CI |
| --- | --- | --- | --- | --- |
| BCL10 | 0.165405 | 0.798671 | 0.581322 | 1.097285 |
| CDC34 | 0.936679 | 1.012992 | 0.736714 | 1.392878 |
| CDC73 | 0.990097 | 0.997993 | 0.726716 | 1.370535 |
| CTR9 | 0.632789 | 1.080185 | 0.787205 | 1.482206 |
| DERL1 | 0.008995 | 1.545349 | 1.114808 | 2.142166 |
| H2BC10 | 0.664728 | 1.072651 | 0.781115 | 1.472998 |
| H2BC11 | 0.569659 | 1.096047 | 0.798964 | 1.503595 |
| H2BC12 | 0.081055 | 0.751296 | 0.544867 | 1.035931 |
| H2BC13 | 0.90591 | 0.98111 | 0.715129 | 1.34602 |
| H2BC14 | 0.614434 | 1.084733 | 0.790482 | 1.488516 |
| H2BC15 | 0.7688 | 1.048748 | 0.763556 | 1.440459 |
| H2BC17 | 0.461275 | 0.887784 | 0.64683 | 1.218497 |
| H2BC3 | 0.114989 | 0.772736 | 0.560786 | 1.064793 |
| H2BC4 | 0.164593 | 0.795974 | 0.576954 | 1.098139 |
| H2BC5 | 0.238819 | 0.826025 | 0.601007 | 1.135291 |
| H2BC6 | 0.869782 | 1.026945 | 0.747295 | 1.411244 |
| H2BC7 | 0.635127 | 0.926152 | 0.674631 | 1.271446 |
| H2BC8 | 0.86336 | 1.028169 | 0.749321 | 1.410786 |
| H2BC9 | 0.927639 | 1.014844 | 0.738399 | 1.394785 |
| HLA-A | 0.118544 | 0.77636 | 0.564964 | 1.066855 |
| HLTF | 0.292476 | 1.1856 | 0.86353 | 1.627791 |
| LEO1 | 0.760065 | 1.050604 | 0.765329 | 1.442213 |
| OTULIN | 0.170767 | 1.248119 | 0.908916 | 1.713911 |
| PAF1 | 0.144201 | 0.78579 | 0.568581 | 1.085976 |
| PCNA | 0.242281 | 1.211777 | 0.878196 | 1.672069 |
| PEX10 | 0.244053 | 0.827698 | 0.602132 | 1.137764 |
| PEX12 | 0.284519 | 0.84087 | 0.612181 | 1.154988 |
| PEX13 | 0.667593 | 1.071969 | 0.780611 | 1.472074 |
| PEX14 | 0.079624 | 0.752638 | 0.547762 | 1.034144 |
| PEX2 | 0.738894 | 1.05531 | 0.768957 | 1.448299 |
| PEX5 | 0.852327 | 0.970349 | 0.706799 | 1.332173 |
| PRKDC | 0.130872 | 1.27909 | 0.929408 | 1.760338 |
| RAD18 | 0.675385 | 1.070219 | 0.778991 | 1.470323 |
| RNF144A | 0.876863 | 0.975265 | 0.710451 | 1.338786 |
| RNF152 | 0.244634 | 1.207377 | 0.878968 | 1.658488 |
| RNF181 | 0.819082 | 1.037652 | 0.755966 | 1.424299 |
| RNF20 | 0.196029 | 1.232767 | 0.897667 | 1.69296 |
| RNF40 | 0.636956 | 0.926232 | 0.67377 | 1.27329 |
| RPS27A | 0.255611 | 0.831249 | 0.604424 | 1.143195 |
| RRAGA | 0.752842 | 0.950342 | 0.69215 | 1.304847 |
| RTF1 | 0.842201 | 0.968313 | 0.705245 | 1.32951 |
| SELENOS | 0.533023 | 1.10578 | 0.806072 | 1.516923 |
| SHPRH | 0.276953 | 1.193928 | 0.867368 | 1.643437 |
| TMEM129 | 0.478076 | 1.121533 | 0.816938 | 1.539694 |
| UBA1 | 0.048926 | 1.37772 | 1.00151 | 1.89525 |
| UBA52 | 0.421086 | 0.877698 | 0.638743 | 1.206046 |
| UBA6 | 0.482266 | 1.120958 | 0.815207 | 1.541383 |
| UBB | 0.999748 | 0.999949 | 0.728881 | 1.371827 |
| UBC | 0.764823 | 0.95274 | 0.693779 | 1.308361 |
| UBE2A | 0.015777 | 1.486214 | 1.077378 | 2.050191 |
| UBE2B | 0.463144 | 1.126001 | 0.820082 | 1.546038 |
| UBE2C | 0.039626 | 1.404834 | 1.016265 | 1.941972 |
| UBE2D1 | 0.352202 | 1.163883 | 0.845386 | 1.602374 |
| UBE2D2 | 0.903965 | 0.98071 | 0.714699 | 1.345731 |
| UBE2D3 | 0.260029 | 1.200516 | 0.873486 | 1.649985 |
| UBE2E1 | 0.59613 | 1.089739 | 0.793041 | 1.497439 |
| UBE2E3 | 0.496986 | 1.117238 | 0.811372 | 1.538408 |
| UBE2G1 | 0.014086 | 1.49847 | 1.084982 | 2.06954 |
| UBE2G2 | 0.263609 | 1.199898 | 0.871734 | 1.651599 |
| UBE2H | 0.976666 | 1.004741 | 0.731839 | 1.379407 |
| UBE2J2 | 0.814952 | 0.962922 | 0.70174 | 1.321313 |
| UBE2K | 0.51973 | 1.109797 | 0.808159 | 1.524018 |
| UBE2L3 | 0.195975 | 1.232528 | 0.897792 | 1.69207 |
| UBE2N | 0.220227 | 1.220829 | 0.887389 | 1.679559 |
| UBE2Q2 | 0.573177 | 1.095293 | 0.798007 | 1.50333 |
| UBE2R2 | 0.366938 | 0.864133 | 0.62923 | 1.186728 |
| UBE2S | 0.847664 | 0.969462 | 0.706489 | 1.33032 |
| UBE2T | 0.007588 | 1.552819 | 1.124136 | 2.144979 |
| UBE2V2 | 0.071445 | 1.345452 | 0.974435 | 1.857735 |
| UBE2W | 0.434624 | 1.135477 | 0.82558 | 1.561699 |
| UBE2Z | 0.472324 | 0.890277 | 0.648449 | 1.22229 |
| UCHL3 | 0.587875 | 0.915617 | 0.665646 | 1.259459 |
| USP5 | 0.90321 | 0.98048 | 0.713599 | 1.347172 |
| USP7 | 0.346765 | 1.164869 | 0.847646 | 1.60081 |
| USP9X | 0.103095 | 1.303858 | 0.947712 | 1.793841 |
| VCP | 0.697909 | 1.064657 | 0.775911 | 1.460857 |
| WAC | 0.040924 | 1.40227 | 1.014045 | 1.939126 |
| WDR61 | 0.991265 | 1.001772 | 0.729685 | 1.375314 |
